# FedPLC: Federated Learning with Dynamic Cluster Adaptation for Concept Drift on Non-IID Data

**DOI:** 10.3390/s26010283

**Published:** 2026-01-02

**Authors:** Qi Zhou, Yantao Yu, Jingxiao Ma, Mohammad S. Obaidat, Xing Chang, Mingchen Ma, Shousheng Sun

**Affiliations:** 1 School of Electronic and Information Engineering, Tongji University, Shanghai 201804, China; 2331824@tongji.edu.cn (Q.Z.); mjxiao@tongji.edu.cn (J.M.); xchang@tongji.edu.cn (X.C.); 2211164@tongji.edu.cn (M.M.); 2310199@tongji.edu.cn (S.S.); 2 School of Computer Science, Shanghai Jiao Tong University, Shanghai 200240, China; 3 Shanghai Key Laboratory of Integrated Administration Technologies for Information Security, Shanghai 200230, China; 4 King Abdullah II School of Information Technology, The University of Jordan, Amman 11942, Jordan; mobaidat@ju.edu.jo; 5 School of Computer and Communication Engineering, University of Science and Technology Beijing, Beijing 100083, China

**Keywords:** federated learning, Non-IID, concept drift, clustering algorithms

## Abstract

In practical deployments of decentralized federated learning (FL) in Internet of Things (IoT) environments, the non-independent and identically distributed (Non-IID) nature of client-local data limits model performance. Furthermore, concept drift further exacerbates complexity and introduces temporal uncertainty that significantly degrades convergence and generalization. Existing approaches, which mainly rely on model-level similarity or static clustering, struggle to disentangle inherent data heterogeneity from dynamic distributional shifts, resulting in poor adaptability under drift scenarios. This paper proposes FedPLC, a novel FL framework that introduces two mechanism-level innovations: (i) Prototype-Anchored Representation Learning (PARL), a strategy inspired by Learning Vector Quantization (LVQ) that stabilizes the representation space against label noise and distributional shifts by aligning sample embeddings with class prototypes; and (ii) Label-wise Dynamic Community Adaptation (LDCA), a fine-grained adaptation mechanism that dynamically reorganizes classifier heads at the label level, enabling rapid personalization and drift-aware community evolution. Together, PARL and LDCA enable FedPLC to explicitly disentangle static Non-IID heterogeneity from temporal concept drift, achieving robust and fine-grained adaptation for large-scale IoT/edge client populations. Our experimental results on the Fashion-MNIST, CIFAR-10, and SVHN datasets demonstrate that FedPLC outperforms the state-of-the-art federated learning methods designed for concept drift in both abrupt drift and incremental drift scenarios.

## 1. Introduction

Federated Learning (FL), as an emerging distributed machine learning framework, is committed to enabling multiple data holders to collaboratively train models without sharing local raw data, providing inherent privacy benefits [1,2,3]. FL effectively aggregates data resources from multiple parties while ensuring data privacy, and has shown great potential in fields such as healthcare, security finance, and e-commerce. Taking e-commerce visual search as an example, FL allows each participant to retain local high-value images and their metadata, and only updates the model parameters through interaction, achieving collaborative optimization of user privacy and model training, thereby improving recommendation quality and user conversion rate. However, the decentralized structure of FL also introduces many complex problems, especially the heterogeneity of data holder distribution, which can be further divided into static Non-IID data and time-varying concept drift.

Non-IID data heterogeneity is an inherent property of the FL participant environment. Due to differences in data collection equipment, user preferences, or business models, data holders often have systematic differences in terms of quantity, feature space, and class label distribution [4,5,6]. This deviation causes different clients to update their local models in different directions, which increases the gradient variance of the global model during the global aggregation process, slows down the convergence rate, and weakens the generalization performance of the model [7,8]. To alleviate the Non-IID problem, existing research focuses on proposing improvement solutions through personalized models or correction aggregation strategies. However, a frequently overlooked challenge is that data distribution dynamically changes over time, especially when the label set itself evolves or expands. Therefore, this paper aims to investigate how to enable the federated model to continuously learn and adapt to the changing labels in the environment.

In contrast, concept drift reflects a higher degree of dynamics and complexity. Its essence is the time-varying nonlinear evolution of the real generation process of local data. Mathematically, it is expressed as the conditional distribution P(Y∣X) evolving asynchronously and heterogeneously over time. Unlike the global concept drift in traditional centralized learning, the concept drift of FL has a distributed characteristic—that is, in the same training round, different clients may be in completely different concept stages; the data distribution of the same client may also undergo multiple local concept updates over time [9]. For example, in e-commerce visual search, the continuous emergence of new product categories, shifting seasonal trends, and evolving user photography devices and habits all cause category annotations or image semantic representations to change synchronously across both client and time dimensions. This highly dynamic and heterogeneous drift pattern seriously threatens the effectiveness of a single global model in addressing all client dynamics. Simple parameter aggregation makes it difficult to find a consistent and effective global solution in this conflicting loss landscape.

Furthermore, the negative impact of concept drift on deep learning model parameters exhibits an inherently hierarchical nature. During the local training phase, gradient descent algorithms drive parameter updates in the representation layers of the model (such as convolutional layers) and in the classification heads (such as fully connected layers) to learn discriminative features from new data modalities. This process fundamentally adjusts the category concepts that characterize the feature space of the model. However, the occurrence of concept drift precipitates conflicting global aggregation updates. As the most exposed component, the classification head experiences an initial catastrophic distortion of its perceptual loss landscape, invalidating the feature-label mapping. This degradation back-propagates, contaminating the gradient signal and corrupting the optimization of the representation layer. Such corruption causes a parameter collapse toward erroneous features, thereby degrading the hierarchical representation. Ultimately, this process distorts the learned category semantic vectors in the feature space, leading to inherent model inconsistencies. We assert that concept drift fundamentally challenges FL by inflicting structural damage on the internal semantic consistency of the model.

Although some existing solutions utilize label-related information, their design goals and technical mechanisms are completely different from this article. FedLC [10] excels in addressing the heterogeneity of traditional static Non-IID data, effectively improving aggregation stability and model convergence speed through its label distribution clustering client design. This work provides important insights into solving classic challenges in federated learning. However, it should be noted that this method is primarily suitable for environments with relatively stable data distributions. When applied to scenarios where data distributions dynamically change over time, its one-off or fixed-period clustering mechanism struggles to capture and adapt to the continuous evolution of the clients’ label space and therefore may not achieve optimal results.

Existing drift-aware FL approaches [11,12] have made significant progress in handling temporal dynamics and can be broadly categorized into three strategic lineages. Single-model adaptation strategies aim to learn a unified global model that is robust to distribution shifts, which is commendable for its simplicity and low communication cost. However, its capacity to handle severe and heterogeneous drifts is often limited, as a single model may struggle to fit all evolving patterns simultaneously. Model decoupling and multi-model adaptation methods offer greater flexibility by maintaining personalized models or sub-networks for clients, effectively capturing diverse local patterns. While these methods excel in personalization, they often lack an explicit mechanism to discern and group clients with similar temporal evolution, potentially leading to inefficient learning and overfitting to local noise. Lastly, clustering-based dynamic adaptation techniques represent a principled effort to group clients based on model similarity or high-level feature distributions, thereby enabling finer-grained aggregation than a single global model. Nevertheless, this reliance on model-level or coarse-grained feature similarities results in a clustering mechanism that is often too coarse to capture fine-grained, label-specific concept drifts. Furthermore, a fundamental challenge persists across these categories: the entanglement of static Non-IID heterogeneity with temporal dynamics is rarely explicitly addressed, which can cause adaptation models to be misled by inherent, time-invariant data biases, thereby degrading the precision of drift detection and adaptation.

To address these limitations, we propose FedPLC, a novel FL framework that explicitly disentangles static heterogeneity and dynamic concept drift through two key mechanisms: (i) Prototype-Anchored Representation Learning (PARL): an LVQ-inspired prototype learning strategy that stabilizes the representation space against label noise and distributional shifts, and (ii) Label-wise Dynamic Community Adaptation (LDCA): a label-wise community detection mechanism that dynamically reorganizes classifier heads at the granularity of individual classes. Together, these components enable FedPLC to achieve robust and fine-grained adaptation under distributed concept drift. The main contributions are as follows:

1. PARL: We propose an LVQ-inspired prototype learning mechanism that decouples the representation layer and the classifier head. Unlike prior work where the representation layer is mainly updated by label-driven supervised signals that can easily become noisy under drift or label corruption, our method introduces prototype alignment losses to anchor representations toward class centers. This design preserves the geometric stability of the feature space even when labels are partially corrupted, enabling the global representation layer to remain robust under sudden or gradual concept drifts. Rather than directly adopting LVQ as a classifier, we reinterpret its prototype competition principle as a mechanism for stabilizing shared representations in federated environments.
2. LDCA: To overcome the limitations of client-level or model-level aggregation, we propose a label-wise dynamic community adaptation mechanism that reorganizes classifier heads at the granularity of individual classes. By constructing client similarity graphs for each label and dynamically detecting communities of clients that share similar discrimination concepts, LDCA enables fine-grained personalization and rapid adaptation when new drift patterns emerge. This prevents concept confusion caused by mixing heterogeneous client distributions and allows communities to evolve dynamically in response to distributed drifts.
3. FedPLC framework: By integrating PARL and LDCA, we present FedPLC, a novel FL framework that explicitly disentangles static statistical heterogeneity and dynamic concept drift. Extensive experiments under extreme Non-IID conditions with both abrupt and incremental drifts show that FedPLC consistently outperforms state-of-the-art baselines, demonstrating the effectiveness and robustness of its mechanism-level innovation.

The rest of this paper is organized as follows: Section 2 reviews related work. Section 3 formally models the research problem. Section 4 elaborates on the proposed FedPLC framework. Section 5 verifies its effectiveness through extensive experiments. Finally, Section 6 concludes the paper.

## 2. Related Work

### 2.1. FL for Non-IID Data

Data heterogeneity is one of the key challenges faced by FL. This heterogeneity may come from many aspects, such as uneven label distribution (label skew), feature distribution difference (feature skew), data quantity disparity (quantity skew) and data quality deviation (quality skew) [13,14]. To address the challenges posed by Non-IID data, the research community has proposed numerous approaches. One type is optimization-based methods, which aim to adjust local model updates to alleviate gradient conflicts caused by data heterogeneity [15]. The effectiveness of these methods is based on a core assumption: the data distribution of the client is static. When the distribution of the real world evolves dynamically over time, these static constraints cannot adapt to the new data pattern in time, resulting in a sharp decline in model performance.

Some studies have taken the perspective of representation learning. In the Non-IID environment, local gradient inconsistency may lead to the collapse of the model representation space (Representation Collapse), that is, features of different categories are mixed in the embedding space. To solve this problem, researchers have proposed enhancing the discriminative power of the representation layer by introducing contrastive loss [16]. Although contrastive learning can effectively reduce aggregation conflicts and enhance the discriminative power of the representation layer, it is highly dependent on the consistency of label-semantics between clients. Once label drift or sudden label errors occur, the label information used to construct positive and negative sample pairs will be contaminated, which will seriously damage the generalization ability and robustness of the model.

Another type of method is personalized federated learning. These methods aim to group clients with similar data distributions into the same clusters and train an independent model for each cluster. This method can provide better performance for clients with specific data distributions. For example, FedHERO [17] leverages a dual-channel graph neural network (GNN) and a structure learner to identify and share structural knowledge across heterogeneous graphs, enhancing model performance. PrivCrFL [11] proposes a cross-cluster FL approach that uses one-shot hierarchical clustering and cross-cluster migration to optimize subgroup convergence. In addition, it introduces intra-cluster learning and inter-cluster learning to enable mutual learning between groups through separate aggregation. However, these clustering methods are usually static. They perform clustering at the beginning of training or at a specific time point but do not take into account that the data distribution of the client will evolve dynamically over time. When concept drift occurs, the fixed clustering structure will become outdated, resulting in performance degradation [18,19]. This shows that relying solely on static clustering to cope with dynamic changes is insufficient, and a method that can dynamically adjust the clustering structure in real time is needed.

### 2.2. Concept Drift Adaptation for FL

In FL, concept drift can occur in the local data of the client, and its form can be divided into virtual concept shift and real concept drift. According to the data distribution, it can be categorized into changes in the input feature distribution P(X), changes in the label prior distribution P(Y), or changes in the category conditional distribution P(Y∣X). These drifts may occur at different speeds (such as abrupt or gradual) and severity. At the same time, concept drift also has unique spatial dimension characteristics in the federated environment. For example, it may only affect some clients (coverage) or occur asynchronously at different time points (synchronism). The combination of data heterogeneity and concept drift, that is, Non-IID data distributed on different clients and dynamically evolving over time, will bring severe challenges to the effectiveness, convergence stability and aggregation mechanism of the model [20]. In terms of dealing with concept drift, there are mainly the following adaptation methods.

#### 2.2.1. Single Model Adaptation Strategy

This type of method aims to use a single global model to adapt to dynamic changes [21,22]. Taking the FLASH [23] algorithm as an example, it detects concept drift by monitoring the amplitude of client model updates and dynamically adjusts the learning rate to accelerate adaptation to new concepts. The core idea is that when drift occurs, the client needs a larger model update to fit the new data distribution. FLASH uses this insight to improve the adaptability of the model. However, this single global model approach has fundamental limitations in dealing with multi-source, distributed drift, because it cannot provide the optimal model for all clients experiencing different drifts at the same time.

#### 2.2.2. Model Decoupling and Multi-Model Adaptation

Another mainstream idea is model decoupling [24], the core idea of which is to divide the model into two parts: a shared representation and a personalized discriminator head. This structure enables the shared part to maintain the common feature extraction capability across clients, whereas the discriminator is responsible for quickly adapting to the local concepts and label deviations of each client. Unlike the contradictory goal of a single global model that attempts to fit all clients simultaneously with a single set of parameters, the decoupled solution explicitly retains personalized paths for multi-source and diverse drift in its structure, and performs better in terms of flexibility and robustness. However, if the label is contaminated or the representation layer needs to be adjusted with drift, how to safely update the representation without destroying global consistency is a key problem.

#### 2.2.3. Clustering-Based Dynamic Adaptation

Clustering federated learning can discover the implicit sub-distribution structure between clients, allowing models to differ between different clusters, thereby achieving a certain degree of personalization [25,26]. Some multi-model methods regard drift adaptation as a time-varying clustering problem. FedDrift [22] hierarchically clusters clients according to their drift patterns and creates a new global model for each new concept. Similarly, FedDAA [27] aims to distinguish real drift from virtual drift through data prototypes to dynamically adjust the number of clusters and adaptation strategies. However, these methods still face challenges in the face of the inherent heterogeneity of Non-IID data. For example, the distance metric used by FedDrift may be misled by the differences between clients caused by static heterogeneity, resulting in incorrect model merging or improper clustering. This makes it difficult to achieve ideal performance in complex scenarios where Non-IID and concept drift coexist.

Limitations of existing research include that most aggregation strategies operate on clients as a whole or the global model, resulting in overly coarse aggregation granularity that masks label-wise differences and induces negative transfer. Furthermore, commonly used similarity metrics based on global parameters or high-level features may fail under concept drift and cannot disentangle static spatial heterogeneity (Non-IID) from dynamic temporal variation (concept drift) from a mechanistic perspective, leading to erroneous clustering and incorrect parameter sharing. This paper introduces PARL and LDCA to decouple and quantify these two types of heterogeneity. This allows FedPLC to focus on addressing real concept drift [28] without being disturbed by inherent statistical differences between clients, thereby achieving more robust and accurate model adaptation.

## 3. Problem Modeling

Consider a personalized FL scenario with a central server and I clients. Each client i∈{1,…,I} maintains a local dataset $D_{i}={\left\{\left(x_{i,n},y_{i,n}\right)\right\}}_{n=1}^{N_{i}}$, where $N_{i}$ denotes the number of samples on client *i*. The data are distributed according to $P_{i}\left(X,Y\right)$, where *X* is the input space and *Y* is the label space containing *C* categories. Each client maintains a personalized model, which consists of a shared representation layer $\theta_{i}$ and a client-specific classifier $\varphi_{i}$. Unlike traditional FL where a single global model is trained, the goal here is to learn models that adapt to the local data distribution of each client, whereas knowledge from other clients is leveraged through prototype-based aggregation and label-wise classifier clustering.

Formally, the local model of client *i* can be written as $\left(\theta_{i},\varphi_{i}\right)$, where $\theta_{i}$ represents the representation parameters and $\varphi_{i}$ denotes the label-wise classifier parameters. Each client optimizes a personalized loss function:

(1) $$L_{i}\left(\theta_{i},\varphi_{i}\right)=L_{\sup}\left(\theta_{i},\varphi_{i};D_{i}\right)+\lambda L_{\mathrm{proto}}\left(\varphi_{i},p_{i}\right),$$

where $L_{\sup}$ is the standard classification loss (e.g., cross-entropy), $L_{\mathrm{proto}}$ aligns the client representation with local and global prototypes $p_{i}$, and λ is a hyperparameter controlling the strength of the prototype alignment.

In FL, the drift of distributed concepts is manifested primarily as the conditional distribution of local client data changing dynamically over time [29], e.g., $P_{i}^{\left(t\right)}\left(Y\;|\;X\right)\neq P_{i}^{\left(t-1\right)}\left(Y\;|\;X\right)$, where *t* represents the communication round. In a distributed scenario, different clients may experience different forms of drift at different time points, e.g., $P_{j}^{\left(t\right)}\left(Y\;|\;X\right)\neq P_{k}^{\left(t-1\right)}\left(Y\;|\;X\right),j\neq k$.

**Figure 1:**
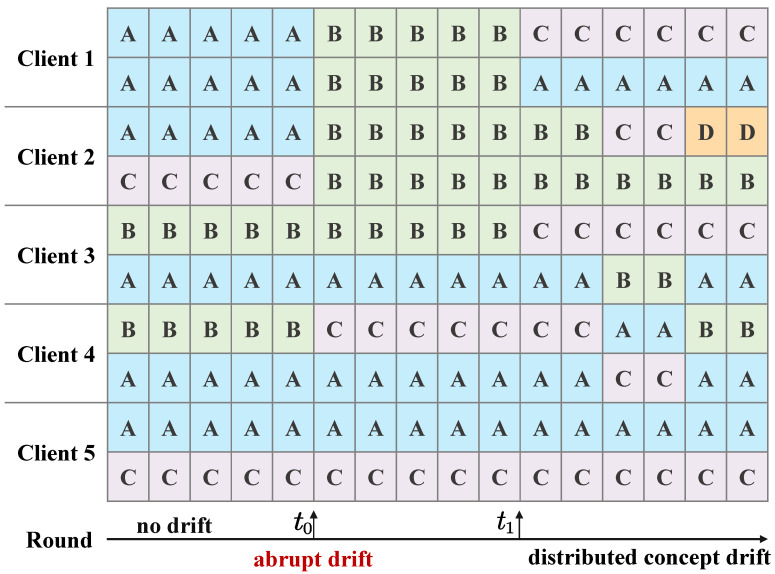
Distributed concept drift representation model. Each colored block denotes the dominant local data concept (A, B, C, or D) observed by a client at a given round. Abrupt drift occurs at round $t_{0}$, and after $t_{1}$, multiple drift forms are mixed.

Figure 1 illustrates the temporal evolution of local data concept states across different clients, covering three representative regimes: a drift-free baseline, an abrupt concept drift, and the most challenging distributed concept drift, thereby highlighting multiple forms of concept drift over successive communication rounds. Each colored block represents the dominant data concept (A, B, C, or D) observed by a client at a given round. In the initial rounds $\left(t<t_{0}\right)$, no concept drift occurs, and the system operates under a drift-free baseline with stable and static core concepts. For example, different clients primarily observe concepts A, B, or C, while these distributions remain stable over time. A single global model, such as the one trained using FedAvg [30], can effectively aggregate knowledge from all clients and achieve strong performance.

At communication round $t_{0}$, abrupt concept drift emerges, where the local data concepts of multiple clients undergo sudden yet asynchronous changes within a single round. Some clients transition from concept A to B, others from concept C to B, while certain clients remain temporarily unchanged. After communication round $t_{1}$, the system evolves into a distributed concept drift regime, in which concept drift manifests in an asynchronous and localized manner. Different clients independently transition among concepts (A, B, C, or D) at different times. No global or coordinated drift pattern exists, leading to pronounced temporal and spatial heterogeneity. Concept drift becomes client-specific and continuously evolving, posing significant challenges to model robustness and consistency in FL. The evolution shown in Figure 1 reflects realistic federated learning scenarios, where data-generating processes may change abruptly and subsequently diverge across clients, underscoring the necessity of adaptive and drift-aware FL frameworks.

This paper mainly considers several typical forms of drift.

### 3.1. Abrupt Drift

At a certain round $t_{0}$, the label distribution of some clients undergoes a sudden change [31], for example, the categories are swapped with:

(2) $$\forall \left(x,y\right)∼P_{i}{\left(X,Y\right)}^{\left(t_{0}-1\right)},\;y^{\prime }=\left\{\begin{array}{cc} a, & y=b,\\ b, & y=a,\\ y, & \mathrm{otherwise}, \end{array}\right.$$

thus obtaining a new conditional distribution $P_{i}^{\left(t_{0}\right)}\left(Y\;|\;X\right)$. The abrupt drift distribution undergoes a drastic jump within a single round, which is manifested as a shift of the prototype:

(3) $$∥p_{i,a}^{\left(t_{0}\right)}-p_{i,a}^{\left(t_{0}-1\right)}∥+∥p_{i,b}^{\left(t_{0}\right)}-p_{i,b}^{\left(t_{0}-1\right)}∥\gg 0.$$

### 3.2. Incremental Drift

In incremental drift, concept drift occurs gradually according to preset stages [32]. For example, different category pairs are exchanged:

(4) $$P_{i}^{\left(t\right)}\left(Y\;|\;X\right)=\left\{\begin{array}{cc} P_{i}^{\left(t_{0}\right)}\left(Y\;|\;X\right), & t_{0}\leq t<t_{1},\\ P_{i}^{\left(t_{1}\right)}\left(Y\;|\;X\right), & t_{1}\leq t<t_{2},\\ \ldots & \ldots \\ P_{i}^{\left(t_{n}\right)}\left(Y\;|\;X\right), & t_{n}\leq t<t_{n+1}, \end{array}\right.$$

which is characterized by a limited drift amplitude each time, but accumulates to form a significant distribution shift over time. Concretely, define a sequence ${\left\{\epsilon_{t}\right\}}_{t=t_{0}}^{t_{n}-1}$ with $\epsilon_{t}\geq 0$, the prototype change satisfies:

(5) $$∥p_{i,c}^{\left(t+1\right)}-p_{i,c}^{\left(t\right)}∥\leq \epsilon_{t},\;\sum_{t=t_{0}}^{t_{n}-1}\epsilon_{t}\gg 0,$$

the single-step perturbation is small, but the long-term accumulation cannot be ignored.

### 3.3. Distributed Drift

The drift occurrence time and category of different clients are different [25]. Formally, suppose the client set I is divided into several subsets $I={⋃}_{m=1}^{M}I_{m},\;I_{j}\cap I_{k}=\emptyset ,\;\forall j\neq k$. Each subset triggers different category exchanges in different rounds. There exists a subset $I_{m}\subseteq I$ such that

(6) $$\begin{array}{c} \forall i\in I_{m}:\;s.t.\;P_{i}^{\left(t\right)}\left(Y\;|\;X\right)=\sigma_{a↔b}\;\left(P_{i}^{\left(t-1\right)}\left(Y\;|\;X\right)\right), \end{array}$$

where $\sigma_{a↔b}$ represents the label exchange operator of category *a* and *b*.

In a Non-IID environment, especially when there is distributed concept drift, a single global model cannot adapt to the local discrimination boundaries of different clients at the same time, which destroys the global consistency assumption and makes it difficult for the global model to adapt to the distribution of all clients at the same time. To this end, the model parameters are decoupled into two parts:

- Shared representation parameters θ, which are uploaded by all clients and aggregated on the server side. The representation is defined as a mapping $f_{\theta }:X\to Z$, $z=f_{\theta }\left(x\right)$, where Z denotes the latent representation space.
- The classification head parameter φ, which connects the deep representation layer, outputs the probability distribution of the class. $f_{\varphi_{i}}:Z\to \Delta^{C-1}$ can be customized for each client.

In this case, the overall goal is to learn the model in a heterogeneous environment with possible concept drift, so as to minimize the expected risk for each client, while ensuring the stability of the representation layer and rapid adaptation to local drift:

(7) $$\min_{\theta ,\left\{\phi_{i}\right\}}\sum_{i=1}^{I}w_{i}E_{\left(x,y\right)∼D_{i}}\left[L_{\sup}\left(h_{\phi_{i}}\left(z\right),y\right)\right]+\lambda L_{\mathrm{proto}}\left(\theta ,\left\{\phi_{i}\right\}\right),$$

where $w_{i}$ is the client weight coefficient (proportional to $N_{i}$), $L_{\sup}$ is the supervision cross entropy, and $L_{\mathrm{proto}}$ is the regularization term used to constrain the alignment of prototype and personalized aggregation.

## 4. Methodology

This paper proposes FedPLC, a novel FL framework designed to jointly address static Non-IID heterogeneity and distributed concept drift. The proposed innovative mechanism, the PARL algorithm, introduces prototype learning inspired by LVQ [33] at the label level, generating representative feature centers for each category, thereby effectively alleviating the model bias and label noise under Non-IID conditions. Meanwhile, LDCA dynamically divides clients with similar data distributions into communities at a fine-grained label level and performs local aggregation within the communities to reduce the adverse effects of concept drift on model performance.

Unlike the single-layer aggregation of traditional FL algorithms, FedPLC adopts an asynchronous two-layer aggregation strategy: First, in each round of communication, the client locally updates model parameters and prototypes. Second, the server detects the dynamic community of the client based on its label distribution and aggregates classifier parameters and community prototypes within the community. Finally, the server broadcasts the aggregation results to the clients in their respective communities. This approach enables efficient local model learning while being robust to data heterogeneity and distribution drift at the global level.

### 4.1. PARL: Prototype-Anchored Representation Learning

Non-IID is the inherent difference in data distribution between clients, whereas real concept drift is the change in the data distribution of a client over time. To decouple these two heterogeneities, the concept of feature prototype inspired by LVQ is introduced, which can well represent the distribution center of each category in the feature space. Therefore, it is a compact and transferable representation of the conditional distribution P(Y∣X) of data, which can reflect the intra-class center and facilitate alignment of supervised representation learning. For a single sample *x*, feature $z=f_{\theta }\left(x\right)$, its true label is $y=\hat{y}$. Classical LVQ update:

(8) $$p_{i,\hat{y}}\leftarrow \left\{\begin{array}{cc} p_{i,\hat{y}}+\eta \left(z-p_{i,\hat{y}}\right) & y=\hat{y}\left(\mathrm{positive}\right),\\ p_{i,{\hat{y}}^{\prime }}-\eta \left(z-p_{i,{\hat{y}}^{\prime }}\right) & y={\hat{y}}^{\prime }\left(\mathrm{negative}\right), \end{array}\right.$$

where $p_{i,\hat{y}}$ denotes the prototype associated with class $\hat{y}$, $p_{i,{\hat{y}}^{\prime }}$ denotes the prototype associated with an incorrect class ${\hat{y}}^{\prime }\neq \hat{y}$, η>0 is the prototype learning rate. For a batch *B*, let the sample feature set of class *c* in the batch be $Z_{i,c}$, then the batch update is: ${\bar{z}}_{i,c}=\frac{1}{\left|Z_{i,c}\right|}\sum_{z\in Z_{i,c}}z$, if $\left|Z_{i,c}\right|>0$ then $p_{i,c}\leftarrow \lambda_{p}p_{i,c}+\left(1-\lambda_{n}\right){\bar{z}}_{i,c},\lambda_{p}\in \left[0,1\right]$, where $\lambda_{p}$ is the prototype retention coefficient. At the same time, a small reverse update is made to the wrong category prototype: if the model prediction of sample *z* is ${\hat{y}}^{\prime }\neq \hat{y}$, then $p_{i,j}\leftarrow p_{i,j}-\eta^{-}\left(z-p_{i,j}\right),\eta^{-}\ll \eta$, where $\eta^{-}$ denotes the reverse learning rate. This term is used to improve the inter-class separation and stay away from negative samples to combat concept confusion.

Inspired by the above LVQ, for a sample $z_{i}=f_{\theta }\left(x_{i}\right)$ and corresponding label $y_{i}$. Let the contrast temperature be τ, and the cosine similarity be $\cos\left(a,b\right)=\frac{a^{⊤}b}{∥a∥∥b∥}$. We define the feature prototype alignment loss $L_{\mathrm{proto}}$ as the contrast InfoNCE algorithm:

(9) $$L_{\mathrm{proto}\;}=-\frac{1}{\left|B\right|}\sum_{i\in B}\log\frac{\exp\left(\cos\left(z_{i},p_{i,y_{i}}\right)/\tau \right)}{\sum_{c=1}^{C}\exp\left(\cos\left(z_{i},p_{i,c}\right)/\tau \right)}.$$

Moreover, an adaptive weight $\lambda_{i}$ is introduced to jointly mitigate static data heterogeneity and adapt to client-specific skewness and concept drift sensitivity. This metric reflects the inherent difference between the data distribution of client *i* and that of the entire federated system in each category. It exists before the drift occurs and is an inherent static property of the client data. The label distribution entropy $H_{i}=-\sum_{c=1}^{C}p_{i,c}\log p_{i,c}$ is used as an indicator to measure the skewness of the static data volume. Then the total loss is defined as:

(10) $$L_{\mathrm{total}}=L_{\sup}+\lambda_{i}L_{\mathrm{proto}\;},\;\lambda_{i}=𝚥H_{i},$$

where $L_{\mathrm{proto}}$ denotes the prototype alignment loss in (9), and *ȷ* is a hyperparameter that regulates the overall strength of prototype alignment. The entropy-based scaling mechanism is designed to modulate the reliability of prototype estimation. Formally, this mechanism prevents clients with highly imbalanced label distributions from being forced to overfit to aggregate prototypes that inadequately represent their data, thereby reducing aggregation-induced deviation.

### 4.2. LDCA: Label-Wise Dynamic Community Adaptation

After successfully decoupling Non-IID and concept drift, the problem is transformed into a dynamic community detection problem.

#### 4.2.1. Client Similarity Graph Construction

In FedPLC, for each label *c*, a client similarity graph $G_{c}=\left(I_{m},E^{\left(c\right)},\varphi \right)$ is constructed. The node set $I_{m}$ represents the clients, and the edge weight $E^{\left(c\right)}$ represents the similarity between the classification heads $\varphi_{c}$ of two clients on label *c*. By ignoring inherent Non-IID differences and focusing on dynamic drift, the community detection algorithm can group clients that truly experience similar changes together, rather than being misled by static data distribution differences between clients. The graph structure is dynamic and is updated at each communication round to capture changes in client relationships.

#### 4.2.2. Classifier-Based Dynamic Community Discovery Algorithm

For each label *c*, LDCA determines which clients have the same concept for the label and perform label-level aggregation within the communities. When the label undergoes concept drift, clients with similar drift will quickly and adaptively cluster into communities and share information, thereby maintaining the diversity of each label. This allows labels to have different community appearances in different clients while reducing the risk of model parameters from other concepts being mixed into the local model. LDCA uses a dynamic community discovery algorithm based on Louvain [34] to perform real-time community partitioning on a graph. Nodes are divided into multiple communities so that the similarity within the community is as high as possible and the similarity between communities is as low as possible. This goal is achieved by maximizing modularity based on the Louvain algorithm.

For a graph $G_{c}$ with total edge weights $2m=\sum_{i,j}e_{ij}$ on label *c*, let the degree of node *i* be $d_{i}=\sum_{j}e_{ij}$. Then, modularity is defined as:

(11) $$Q=\frac{1}{2m}\sum_{i,j}\left(e_{ij}-\frac{d_{i}d_{j}}{2m}\right)\delta \left(g_{i},g_{j}\right),$$

where $\delta (g_{i},g_{j})$ is the Kronecker delta, that is equal to 1 when nodes *i* and *j* belong to the same community and 0 otherwise. The term $\frac{d_{i}d_{j}}{2m}$ represents the expected edge weight between nodes *i* and *j* in a random network with the same degree distribution. A larger difference between the observed edge weight $e_{ij}$ and the expected value indicates that the connections within the community are tighter than those in a random network. Maximizing *Q* thus yields the partition that best captures the community structure.

This dynamic clustering solves the problem of model merging errors and contamination mentioned in FedDrift by decoupling the confusion caused by Non-IID. It addresses challenges that neither single global models nor static clustering models can solve. It achieves a balance between model personalization (classification head) and generalization (intra-community collaboration), enabling the global model to quickly adapt to evolving concepts. Each node forms a community by itself, and local greedy optimization is performed. Iterate over each node *i* and calculate the modularity gain ΔQ when moving it to the neighboring community $G_{c}$. If the gain is positive, perform the move:

(12) $$\begin{array}{cc} \Delta Q & =\frac{1}{2m}\left[\sum_{\mathrm{in}}+2d_{i,\mathrm{in}}-\frac{{\left(\sum_{\mathrm{tot}}+d_{i}\right)}^{2}}{2m}\right]\\ \; & \;-\frac{1}{2m}\left[\sum_{\mathrm{in}}-\frac{{\left(\sum_{\mathrm{tot}}\right)}^{2}}{2m}-\frac{d_{i}^{2}}{2m}\right], \end{array}$$

where $\sum_{\mathrm{in}}$ is the sum of the internal edge weights of community $G_{c}$ (excluding node *i*), $\sum_{\mathrm{tot}}$ is the total degree of community $G_{c}$ (excluding node *i*), and $d_{i,\mathrm{in}}$ is the sum of the edge weights between node *i* and community $G_{c}$.

The expression can be simplified as $\Delta Q=\frac{d_{i,\mathrm{in}}}{m}-\frac{d_{i}\sum_{\mathrm{tot}}}{2m^{2}}$, if ΔQ>0, then move *i* into the community. Treat each community as a super node and reconstruct the new graph. The edge weight is the sum of the edge weights between the communities in the original graph. Repeat the above steps until the modularity *Q* converges or the gain is less than the threshold.

The global models of different communities remain independent, thereby achieving parallel adaptation to different drift concepts. This multi-classification head scheme avoids the performance bottleneck of the global classification head in the decentralized drift scenario. In addition, through PARL and LDCA, FedPLC solves the challenges of how to determine the number of communities and how to effectively cluster in the multi-model method. Within each community, the server performs label-wise aggregation:

(13) $${\bar{\varphi }}_{c}=\frac{\sum_{j\in G_{c}}\left|D_{j,c}\right|\varphi_{j,c}}{\sum_{j\in G_{c}}\left|D_{j,c}\right|},{\bar{p}}_{c}=\frac{\sum_{j\in G_{c}}\left|D_{j,c}\right|p_{j,c}}{\sum_{j\in G_{c}}\left|D_{j,c}\right|}.$$

Each client obtains the aggregated label-level fine-grained parameters as (13) from its community, including community prototypes and classifiers for the next round of training. In this way, community detection acts as a filter, where homogeneous clients share the aggregation within the community, and differences are maintained between communities to avoid false mixing.

PARL and LDCA work together to explicitly decouple static statistical heterogeneity from dynamic distribution drift in a distributed setting, thereby achieving robust and fine-grained adaptability.

### 4.3. FedPLC Algorithm Process

The FedPLC process is formally presented in Algorithm 1. All clients initialize their local models. In each round *t*, each selected client i∈I first performs warm-up training on the classifier (lines 4–6). Lines 7–8 perform decoupled local training, training the local classifier and representation layer, respectively. Lines 9–11 compute feature prototypes. After receiving the representation layer parameters, classifier parameters, and feature prototypes, the server initiates the aggregation process. Lines 13–14 and 15–16 correspond to representation layer aggregation and feature prototype aggregation. Lines 17–19 and 20 correspond to label-level classifier graph construction and community detection algorithms. Lines 23–26 aggregate the personalized local classifiers and community prototypes for each formed community and update the corresponding parameters of the community.
**Algorithm 1** FedPLC**Input:** Total number of rounds *T*, initial model {θ,φ}, representation LR $\eta_{\theta }$, classifier LR $\eta_{\varphi }$, local epochs *E*, warm-up iters *B*, community detection distance threshold ζ**Output:** personalized model $\left\{\theta_{i}^{\left(E\right)},\varphi_{i}^{\left(E\right)}\right\}$, Accuracy $A_{\mathrm{global}}$  1:Broadcast $\left\{\theta^{\left(0\right)},\varphi^{\left(0\right)}\right\}$ to all clients  2:**for** t=1 to *T* **do**  3:   Initialize ${\left\{i\right\}}^{\left(0\right)}=\mathrm{RandomSelect}\left(I,M\right)$  4:   **for** $i^{\left(t\right)}\in \left\{1,2,\ldots ,M\right\}$ **do**  5:     Warm-up on $\left(M,B,D_{i,\mathrm{warm}-\mathrm{up}}^{\left(t\right)}\right)$ with fixed $\theta^{\left(0\right)}$:  6:        $\varphi_{i}^{\left(b\right)}\leftarrow \varphi_{i}^{\left(0\right)}-\eta_{\varphi }\nabla_{\varphi }L\left(\theta_{i}^{\left(0\right)},\varphi_{i}^{\left(0\right)}\right)$  7:     Fix $\theta_{i}$, train $\varphi_{i}$ for $E_{\varphi }$ epochs  8:     Fix $\varphi_{i}$, train $\theta_{i}$ for $E_{\theta }$ epochs following (10)  9:     **for** c∈{1,…,C} **do**10:        $p_{i,c}^{\left(t\right)}=\frac{1}{|D_{i,c}^{\left(t\right)}|}\sum_{\left(x,c\right)\in D_{i,c}^{\left(t\right)}}f_{\theta_{i}^{\left(t\right)}}\left(x\right)$11:     **end for**12:   **end for**13:   Aggregate representation parameters:14:      $\theta^{\left(t+1\right)}\leftarrow \frac{\sum_{i\in M^{\left(t\right)}}\left|D_{i}^{\left(t\right)}\right|\theta_{i}^{\left(t\right)}}{\sum_{i\in M^{\left(t\right)}}\left|D_{i}^{\left(t\right)}\right|}$15:   Aggregate global prototypes:16:      ${\bar{p}}_{c}^{\left(t\right)}\leftarrow \frac{\sum_{i\in M^{\left(t\right)}}\left|D_{i,c}^{\left(t\right)}\right|p_{i,c}^{\left(t\right)}}{\sum_{i\in M^{\left(t\right)}}\left|D_{i,c}^{\left(t\right)}\right|}$17:   **for** c∈{1,…,C} **do**18:     ${\left\{\varphi_{i,c}=\left[W_{i,c}^{\left(\varphi \right)},b_{i,c}^{\left(\varphi \right)}\right]\right\}}_{i=1}^{M}$19:     $G_{c}=\left(I_{m},E^{\left(c\right)},\varphi \right)$, $E=\{{\left(i,j\right)}^{\left(c\right)}\;|\;{\mathrm{sim}}_{i,j}^{\left(c\right)}>\zeta \}$20:     $C^{\left(c,t\right)}\leftarrow \mathrm{Louvain}\left(G_{c},\delta \right)$ following (12)21:   **end for**22:   **for** $C_{k}^{\left(c,t\right)}\subseteq C^{\left(c,t\right)}$ **do**23:     **if** $|C_{k}^{\left(c,t\right)}|>1$ **then**24:            ${\bar{\varphi }}_{k,c}^{\left(t\right)}\leftarrow \frac{\sum_{j\in C_{k}^{\left(c,t\right)}}\left|D_{j,c}^{\left(t\right)}\right|\;\varphi_{j,c}^{\left(t\right)}}{\sum_{j\in C_{k}^{\left(c,t\right)}}\left|D_{j,c}^{\left(t\right)}\right|}$25:            ${\bar{p}}_{k,c}^{\left(t\right)}\leftarrow \frac{\sum_{j\in C_{k}^{\left(c,t\right)}}\left|D_{j,c}^{\left(t\right)}\right|\;p_{j,c}^{\left(t\right)}}{\sum_{j\in C_{k}^{\left(c,t\right)}}\left|D_{j,c}^{\left(t\right)}\right|}$26:     **end if**27:   **end for**28:**end for**

## 5. Performance Evaluation

Horizontal federated learning (HFL) primarily addresses the problem of data silos and was originally designed for cross-device collaborative training scenarios [35]. HFL focuses on joint learning over different subsets of samples within a shared feature space, which constitutes a key distinction from vertical federated learning (VFL) and federated transfer learning (FTL) [36]. This paper adopts the HFL paradigm, where clients share a common feature space; however, due to statistical heterogeneity and Non-IID data partitioning, the label distributions observed by different clients may vary substantially, often manifesting as partially overlapping label subsets. Specifically, each client holds locally collected image–label pairs with identical input modalities and task definitions, while the data are partitioned across clients by samples rather than by features. This setting aligns with practical large-scale vision applications such as e-commerce visual search, where multiple participants collect heterogeneous yet structurally homogeneous data streams over time.

We uses Python 3.12.7 and torch components to design a simulation environment for the performance evaluation of FedPLC and other solutions. The experimental setup is equipped with an Intel(R) i7-12650H processor and an RTX 4060 graphics card. We evaluate FedPLC on three benchmark datasets: Fashion-MNIST [37], CIFAR-10 [38], and SVHN [39]. These datasets are standard image classification benchmarks widely adopted in federated learning and concept drift research, which allows fair comparison with prior work. It is worth noting that the proposed mechanisms (PARL and LDCA) are domain-agnostic and do not rely on image-specific properties; thus, image datasets are chosen purely as representative benchmarks rather than application constraints. To ensure fairness and reproducibility, all methods use the same model architecture and training configuration.

Dataset Description: This paper uses three classic image classification benchmark datasets for experimental evaluation: Fashion-MNIST [37], CIFAR-10 [38], and SVHN [39]. Fashion-MNIST contains 70,000 grayscale images of size 28 × 28, with 60,000 training samples and 10,000 test samples, covering 10 clothing categories. CIFAR-10 consists of 60,000 color images of size 32 × 32 × 3 (RGB, 50,000 for training and 10,000 for testing) across 10 common object classes such as airplanes, cars, and animals. SVHN is collected from real-world street view images and includes about 73,000 training samples and 26,000 test samples, with images of size 32 × 32 × 3 representing digits from 0 to 9. Compared to Fashion-MNIST and CIFAR-10, SVHN has higher background complexity and noise levels, more closely resembling the non-ideal data distribution scenarios of the real world. Collectively, these three datasets are representative in terms of sample size, feature dimension, and data complexity, helping to comprehensively evaluate the robustness and generalization ability of FedPLC in heterogeneous federated learning environments from multiple perspectives.

**Figure 2:**
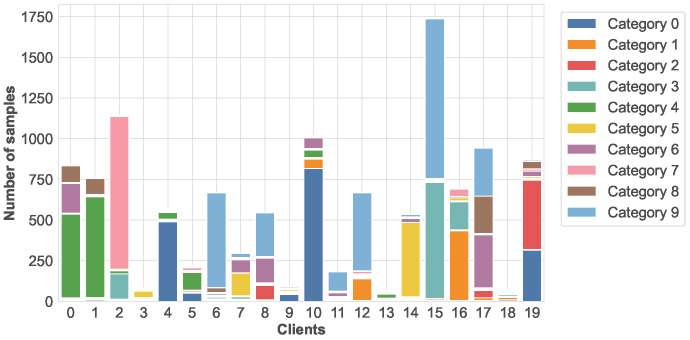
Data distribution of 20 randomly selected clients in a round.

Non-IID Simulation: To simulate statistical heterogeneity across clients, a Dirichlet distribution [40] is adopted to partition the data, where a smaller concentration parameter α leads to a higher degree of Non-IID heterogeneity. Unlike modeling methods that only focus on class imbalance or data volume imbalance, the Dirichlet distribution can jointly model class distribution skewness and data volume heterogeneity within a unified probabilistic framework. This data partitioning method more accurately reflects the multidimensional complexity of data distribution in real-world environments, and is particularly well-suited to the highly heterogeneous and distributed nature of terminal device data in IoT environments. Under this setting, each client receives a skewed label distribution, resulting in substantial variability in both label diversity and sample volume across clients. Figure 2 illustrates the local data distribution of 20 clients randomly selected from a total of 100 clients in a randomly chosen training round on the Fashion-MNIST dataset. As shown in the figure, the degree of class imbalance varies significantly across clients. Some clients have samples primarily concentrated in a single class, with several hundred samples belonging to that class and only a small number of samples from other classes. Other clients contain samples from two or more classes, where one or two classes dominate, while the non-dominant classes are still non-negligible. In contrast, a few clients exhibit relatively more balanced yet still highly skewed distributions. This imbalance reflects the real-world FL scenario in which data are generated according to user-specific preferences or usage patterns, and individual clients may only observe a limited subset of labels. Such heterogeneous data distributions pose significant challenges for global model convergence and generalization, thereby providing a rigorous testbed for evaluating the robustness and personalization capability of FedPLC under severe Non-IID conditions.

Concept Drift Simulation: For each client, the label switching rules are as follows:

(14) $$\sigma_{i}=\left\{\begin{array}{cc} \sigma_{1↔2}, & \mathrm{if}\;(i\;\mathrm{mod}\;10)<3,\;\mathrm{Global}\;\mathrm{ID}=1,\\ \sigma_{3↔4}, & \mathrm{if}\;3\leq (i\;\mathrm{mod}\;10)<6,\;\mathrm{Global}\;\mathrm{ID}=2,\\ \sigma_{5↔6}, & \mathrm{if}\;(i\;\mathrm{mod}\;10)\geq 6,\;\mathrm{Global}\;\mathrm{ID}=3. \end{array}\right.$$

The experiment simulated two types of conditional distribution changes over time:

- Abrupt Drift: At a specific training epoch, the data label distribution for some clients was replaced with a new, completely different distribution. This was set at epoch 100.
- Incremental Drift: Different clients experienced different concept drifts at different points in time to simulate the temporal and spatial heterogeneity of real-world data changes. This was set at epochs 100, 120, and 140.

Baseline Models: Representative algorithms in this field were selected for comparison, including:

- Drift-independent algorithms: FedAvg [30], FedProx [41], and FedFM [42].
- Drift-aware algorithms: Adaptive-FedAvg [43], pFedMe [12], Ditto [44], FedRep [45], FedBABU [24], FedPAC [46], FedDrift [22], IFCA [47], and FLASH [23].

Implementation Details: A comprehensive evaluation of generalization performance and drift adaptability was conducted. All experiments were run for 200 communication rounds, with each client reporting the generalized accuracy of its model across the entire test set, and the average evaluation accuracy was recorded. To ensure reproducibility, a fixed random seed was set so that the conditional distribution of the training and test sets of each client remained consistent across epochs. Local training employed the SGD optimizer with weight decay of $1\times 10^{-5}$ and momentum of 0.9. The number of local epochs was set to E=5 for all datasets. For the decoupled methods, the learning rates were $\eta_{\theta }=0.01$ and $\eta_{\varphi }=0.1$, whereas for the other methods the local learning rate was fixed at 0.01. For FedPLC, the similarity threshold was ζ=0.8, the entropy scaling factor as (10) was *ȷ* = 0.05, the classification head pre-training iterations were 5, and the batch size was set to 3 samples per class.

### 5.1. Performance in Drift-Free Scenarios

**Figure 3:**
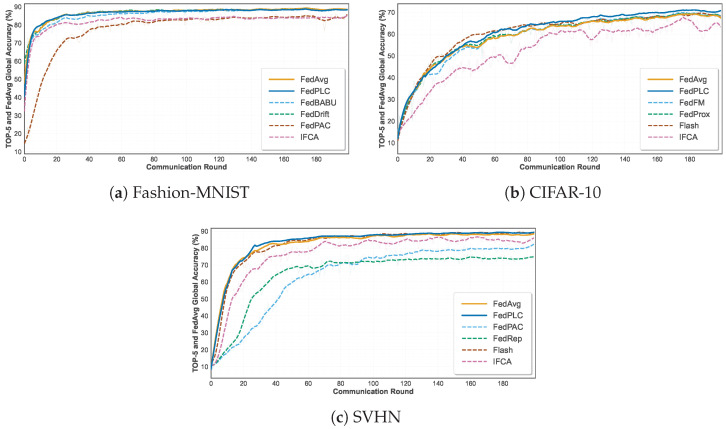
Drift-free comparison on various datasets.

This experiment first systematically evaluates multiple federated learning algorithms in a drift-free benchmark setting to establish performance baselines for each method under an ideal data distribution and analyze their inherent optimization capabilities. Figure 3 shows the global accuracy of the top six methods including the FedAvg baseline on the three datasets under the drift-free benchmark setting, whereas Table 1 summarizes the detailed accuracy results of all methods. Specifically, on Fashion-MNIST, FedPLC (88.38%) is roughly comparable to mainstream methods such as FedAvg (88.97%) and FedProx (89.03%). On SVHN (89.09%), it is also similar to FedFM (89.11%) and AdapFedAvg (89.32%), demonstrating that our approach does not sacrifice model accuracy in the absence of concept drift. On CIFAR-10, FedPLC achieves 70.30%, surpassing all baselines.

Experimental results demonstrate that FedPLC achieves significant performance improvements on complex image datasets such as CIFAR-10, while maintaining highly competitive accuracy on other datasets. This finding preliminarily validates that the optimization strategy PARL employed by FedPLC is an effective representation learning mechanism that enhances the discriminative power of the global model representation, thereby improving its generalization ability in stable environments.

The results further reveal performance differences between algorithms: Algorithms focused on client-side personalization (such as Ditto and FedRep) generally exhibit lower global accuracy, demonstrating the inherent trade-off between personalization and global generalization. In contrast, FedPLC achieves improvements while maintaining global performance. This is primarily due to its prototype alignment mechanism PARL, which enables the co-optimization of client-side local models and global models in the discriminative feature space, resulting in a more robust and generalizable global model. These baseline experiments provide a critical comparison for subsequent performance analysis in concept drift scenarios.

### 5.2. Performance in Abrupt Drift Scenarios

**Figure 4:**
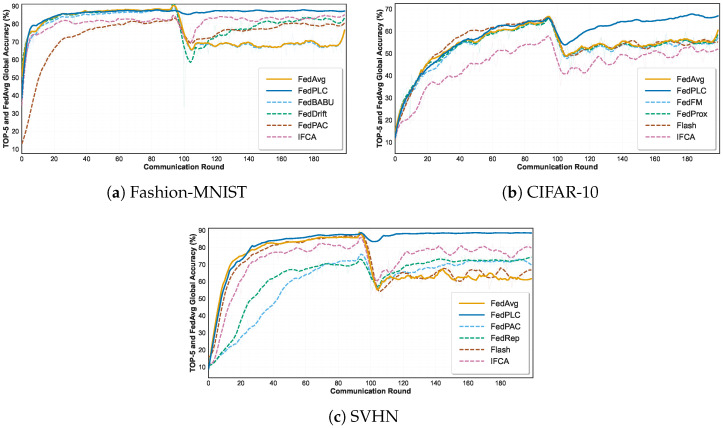
Abrupt drift comparison on various datasets.

FedPLC significantly outperforms all baseline methods on the Fashion-MNIST, CIFAR-10, and SVHN datasets under abrupt drift settings. As shown in Figure 4 and Table 2, when concept drift occurs mid-training, the accuracy of all drift-independent algorithms drops dramatically. Drift-aware algorithms such as FedDrift and FLASH can recover performance to some extent, whereas their recovery is slower and their ultimate accuracy is lower than that of our approach. FedPLC, with its unique decoupling and community detection mechanisms, can immediately identify drifting clients and create new communities and models for them, enabling fast and efficient adaptation.

**Figure 5:**
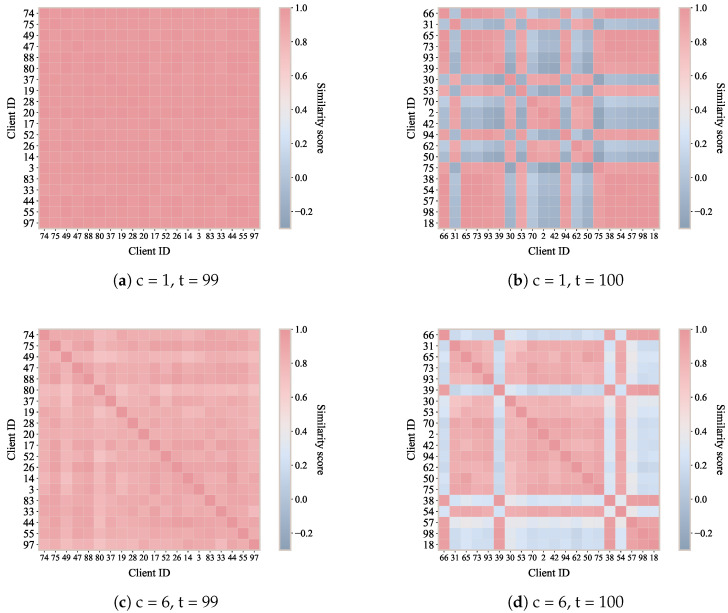
Label-wise Dynamic Community Adaptation on Fashion-MNIST: similarity heatmap of different labels for 20 randomly sampled clients.

As shown in Figure 5, the first row shows the label 1 community detection results for 99 rounds (Figure 5a, drift-free) and 100 rounds (Figure 5b, abrupt drift). Each element (*i*, *j*) in the matrix represents the similarity value between client *i* and client *j*. The other row (Figure 5c,d) shows the corresponding similarity heatmaps for label 6 community detection.

In federated environments, a change or expansion of the local label set fundamentally alters the semantic mapping between feature representations and classifier outputs, making model modification unavoidable. When new labels emerge or existing labels shift in meaning, the original classification head—trained under a previous label configuration—no longer provides a valid decision boundary for the updated data distribution. As illustrated in Figure 5, such changes manifest as abrupt variations in inter-client similarity at the classifier-head level, even when the underlying feature extractor remains partially stable. If the model structure were kept fixed, these semantic inconsistencies would propagate backward, contaminating representation learning and leading to degraded global aggregation. FedPLC explicitly addresses this issue by allowing classifier heads to be dynamically reorganized at the label level: before abrupt drift, clients remain statically grouped based on their Non-IID characteristics, maintaining a stable community structure. When some clients experience concept drift and label-wise similarity patterns change, FedPLC quickly detects the change in their classification heads and reassigns affected clients to new communities. It then trains personalized classification heads for the new concepts, guiding the training of feature prototypes and the global representation layer. This dynamic clustering based on change patterns rather than inherent differences is the key to the robustness of FedPLC in complex environments.

### 5.3. Performance in Incremental Drift Scenarios

**Figure 6:**
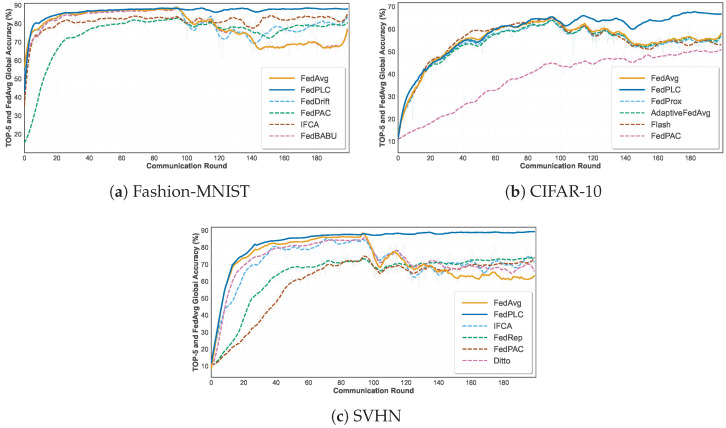
Incremental drift comparison on various datasets.

As shown in Figure 6 and Table 3, traditional federated optimization methods such as FedAvg and FedProx achieve moderate accuracy under incremental drift, but their performance degrades rapidly in the face of severe distribution shifts.

Personalized methods (e.g., pFedMe and Ditto) demonstrate improved robustness, especially on Fashion-MNIST and SVHN, suggesting that local adaptation can mitigate drift to some extent. Clustering-based methods such as IFCA also outperform most baselines by capturing heterogeneous client behavior. However, their accuracy remains limited on challenging datasets such as CIFAR-10.

Notably, the proposed FedPLC consistently achieves the best results on all three benchmarks. Specifically, it improves the average test accuracy to 87.86% on Fashion-MNIST, 66.66% on CIFAR-10, and 89.09% on SVHN, outperforming the best-performing baselines (IFCA and FedPAC) by 5.36%, 16.01%, and 15.49%, respectively. This significant improvement demonstrates that by integrating PARL and LDCA, FedPLC effectively maintains a stable decision boundary even in the face of Non-IID and distributed incremental drift. The consistent improvement across datasets further demonstrates its strong generalization ability and adaptability to dynamic environments.

### 5.4. Ablation Study on the Community Detection Distance Threshold ζ

To investigate the sensitivity of FedPLC to the community detection distance threshold ζ used in LDCA, we conduct an ablation study varying ζ from 0.6 to 0.95. This threshold controls the minimum similarity required for clients to be grouped into the same label-wise community, thereby directly affecting the granularity of dynamic community formation. A lower threshold encourages aggressive merging of heterogeneous clients, while a higher threshold leads to finer-grained but potentially over-fragmented communities. We evaluate the final test accuracy of FedPLC under abrupt drift settings on Fashion-MNIST, CIFAR-10, and SVHN.

**Figure 7:**
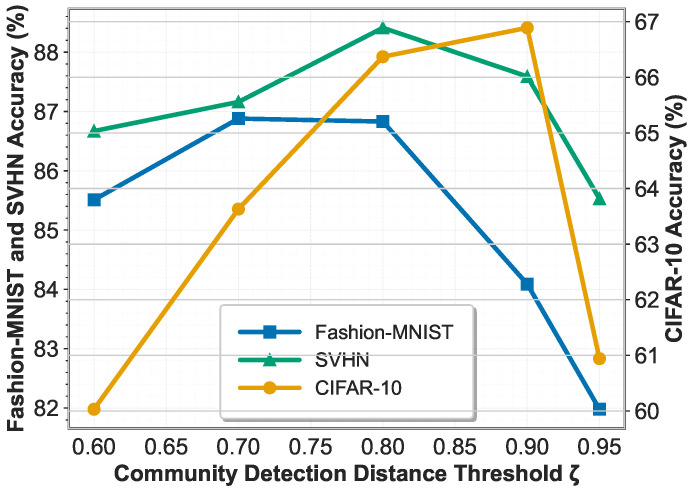
Ablation study on distance threshold ζ. ζ = 0.8 is an optimal selection.

Figure 7 illustrates the impact of different community detection thresholds on model performance. When the threshold is too low, different clients are prematurely merged into the same community, especially in the case of concept drift. This leads to excessive interference between heterogeneous classifiers, thus reducing accuracy, which is particularly evident on the CIFAR-10 dataset. Conversely, when the threshold is too high, the community structure becomes overly fragmented. While this reduces negative transfer, it limits effective information sharing and weakens global coordination, resulting in a significant performance drop across all datasets. Overall, FedPLC performs best when the threshold is set in a moderate range (0.8–0.9), indicating a good balance between community stability and adaptive flexibility.

Notably, CIFAR-10 is most sensitive to threshold selection, reflecting its complex visual semantics and high inter-class confusion. In contrast, Fashion-MNIST and SVHN are relatively more robust but exhibit the same trend. These results confirm that the community detection threshold plays a crucial role in balancing individualization and collaboration in FedPLC.

## 6. Conclusions

In summary, FedPLC disentangles static Non-IID heterogeneity and dynamic concept drift through PARL and LDCA, achieving robust and fine-grained adaptation in federated environments. PARL stabilizes the shared representation space by anchoring sample embeddings to LVQ-inspired class prototypes, mitigating representation collapse under label or feature distribution shifts. LDCA performs fine-grained, label-wise community detection and aggregation of classifier heads, enabling rapid, localized personalization under asynchronous, label-specific drift. Extensive experiments on multiple benchmarks validate its effectiveness and superiority over state-of-the-art methods.

While the proposed PARL demonstrates significant potential in concept representation learning by leveraging label consistency to build prototypes, its performance heavily relies on high-quality data annotation. However, in real-world IoT deployment environments, data is typically subject to significant noise interference, and manual annotation inevitably introduces labeling errors. An important future research direction is exploring ways to enhance the robustness of such methods. Specifically, future work could focus on developing robust prototype learning mechanisms insensitive to noise and labeling errors. For example, this could involve introducing uncertainty quantification to assess prototype confidence or designing noise-tolerant contrastive learning objectives, thereby ensuring model stability in environments with fluctuating data quality. Another promising direction for future research is to explore the scalability of FedPLC in large-scale multimodal networks where different modalities may emerge, disappear, or evolve over time. Extending FedPLC to handle asynchronous modality-specific drift and inconsistent semantic granularity will help develop more general and adaptive federated learning systems capable of operating in complex real-world environments.

## Figures and Tables

**Table 1:** Generalized accuracy for no concept drift under a DIR (0.1). For 100 customers, the sample ratio is 20%. All results are the average of the three test set accuracy (%) ± STD.

Method	Fashion-MNIST	CIFAR-10	SVHN
AdapFedAvg	89.37 ± 0.28	67.82 ± 0.68	89.32 ± 0.32
FedAvg	88.97 ± 0.35	67.09 ± 0.20	88.62 ± 0.12
FedProx	89.03 ± 0.07	67.90 ± 0.22	88.64 ± 0.35
FedFM	88.39 ± 0.28	68.03 ± 0.34	89.11 ± 0.79
Flash	88.71 ± 0.30	68.07 ± 0.25	88.92 ± 0.25
Ditto	87.84 ± 0.21	64.52 ± 0.45	86.57 ± 0.39
FedBABU	77.46 ± 0.65	48.32 ± 0.23	70.30 ± 0.28
FedDrift	88.61 ± 0.35	67.78 ± 0.75	88.33 ± 0.77
FedPAC	84.08 ± 0.40	56.61 ± 0.40	80.16 ± 0.38
FedRep	76.91 ± 0.30	45.63 ± 1.42	73.69 ± 0.13
IFCA	85.12 ± 0.56	62.64 ± 0.30	85.68 ± 0.38
pFedMe	83.02 ± 0.29	49.75 ± 0.81	74.06 ± 0.50
FedPLC	88.38 ± 0.22	70.30 ± 0.27	89.09 ± 0.68

**Table 2:** Generalized accuracy for abrupt concept drift under a DIR (0.1). For 100 customers, the sample ratio is 20%. All results are the average of the three test run accuracy (%) ± STD.

Method	Fashion-MNIST	CIFAR-10	SVHN
AdapFedAvg	68.18 ± 0.45	52.91 ± 0.31	64.23 ± 0.25
FedAvg	68.53 ± 1.77	55.09 ± 0.25	62.37 ± 0.39
FedProx	67.97 ± 0.20	53.79 ± 0.11	65.12 ± 0.55
FedFM	67.19 ± 0.55	53.70 ± 0.42	65.68 ± 1.29
Flash	68.00 ± 0.98	54.30 ± 1.11	66.43 ± 1.55
Ditto	73.16 ± 0.28	52.64 ± 0.16	68.66 ± 0.34
FedBABU	77.90 ± 0.15	45.18 ± 0.25	69.01 ± 0.40
FedDrift	81.30 ± 0.04	46.13 ± 0.36	58.39 ± 1.43
FedPAC	79.46 ± 0.42	49.73 ± 1.21	71.16 ± 0.48
FedRep	77.39 ± 0.29	46.23 ± 1.56	72.94 ± 0.14
IFCA	83.88 ± 0.21	53.00 ± 0.48	78.76 ± 2.14
pFedMe	71.59 ± 0.18	40.97 ± 0.47	59.25 ± 0.34
FedPLC	86.83 ± 0.13	66.37 ± 0.71	88.41 ± 0.45

**Table 3:** Generalized accuracy for incremental drift under a DIR (0.1). For 100 customers, the sample ratio is 20%. All results are the average of the three test set accuracy (%) ± STD.

Method	Fashion-MNIST	CIFAR-10	SVHN
AdapFedAvg	68.27 ± 0.37	53.84 ± 0.18	61.67 ± 0.55
FedAvg	68.28 ± 0.90	53.97 ± 0.47	64.22 ± 0.33
FedProx	67.72 ± 0.29	53.14 ± 0.43	64.66 ± 0.42
FedFM	66.62 ± 0.42	52.16 ± 0.49	62.86 ± 0.76
Flash	67.58 ± 0.13	52.41 ± 0.22	65.72 ± 0.13
Ditto	72.98 ± 0.20	52.49 ± 0.21	68.72 ± 0.18
FedBABU	77.11 ± 0.49	45.84 ± 0.66	66.96 ± 0.58
FedDrift	80.35 ± 0.30	43.45 ± 2.21	54.99 ± 0.23
FedPAC	79.66 ± 0.31	50.49 ± 0.37	72.00 ± 0.26
FedRep	76.61 ± 0.17	45.83 ± 0.59	73.60 ± 0.36
IFCA	82.50 ± 0.29	50.65 ± 0.23	71.39 ± 0.36
pFedMe	71.16 ± 0.24	40.96 ± 0.56	60.25 ± 0.82
FedPLC	87.86 ± 0.23	66.66 ± 0.28	89.09 ± 0.46

## Data Availability

The original contributions presented in this study are included in the article. Further inquiries can be directed to the corresponding author.
